# Oral administration of marine collagen peptides prepared from chum salmon (*Oncorhynchus keta*) improves wound healing following cesarean section in rats

**DOI:** 10.3402/fnr.v59.26411

**Published:** 2015-05-13

**Authors:** Junbo Wang, Meihong Xu, Rui Liang, Ming Zhao, Zhaofeng Zhang, Yong Li

**Affiliations:** 1 Department of Nutrition and Food Hygiene, School of Public Health, Peking University, Beijing, China; 2 Beijing Key Laboratory of Toxicological Research and Risk Assessment for Food Safety, Peking University, Beijing, China

**Keywords:** cesarean section, marine collagen peptide, wound healing, basic fibroblast growth factor, transforming growth factor beta 1, CD31

## Abstract

**Background:**

The goal of the present study was to investigate the wound-healing potential of marine collagen peptides (MCPs) from chum salmon skin administered to rats following cesarean section (CS).

**Methods:**

Ninety-six pregnant Sprague-Dawley rats were randomly divided into four groups: a vehicle group and three MCP groups. After CS, rats were intragastrically given MCPs at doses of 0, 0.13, 0.38, 1.15 g/kg*bw, respectively. On postoperative days 7, 14, and 21, the uterine bursting pressure, skin tensile strength, hydroxyproline (Hyp) concentrations, and histological and immunohistochemical characteristics of the scar tissue were examined.

**Results:**

In the MCP groups, the skin tensile strength, uterine bursting pressure, and Hyp were significantly higher than those in the vehicle group at all three time points (*p*<0.05). The formation of capillary, fibroblast, and collagen fiber, the expression of platelet-endothelial cell adhesion molecule-1, basic fibroblast growth factor, and transforming growth factor beta-1 were increased in the MCP groups (*p*<0.05).

**Conclusion:**

MCPs could accelerate the process of wounding healing in rats after CS.

Caesarean section (CS) is currently the most common abdominal surgical procedure performed globally. CS rates have continued to increase over the past two decades, and this upward trajectory appears likely to continue in the near future (1–4). As an alternative procedure for child delivery, however, CS is an invasive and risk-bearing medical practice for both maternal and neonatal health (3, 4). Wound complications are a major source of morbidity after CS and contribute to prolonged hospital stay and readmission (5). Many women undergoing caesarean delivery develop wound complications postoperatively, including seroma, hematoma, wound infection, wound separation, and wound dehiscence (6, 7). Furthermore, incomplete healing of uterine incision after CS also is an etiologic factor in many clinical problems, such as the rupture of the uterus during a subsequent pregnancy, ectopic pregnancy at the CS scar, abnormal uterine bleeding, and dysmenorrhea during the non-pregnant state (8). Many techniques have been investigated to decrease wound complications, including surgical strategy, skin closure methods, subcutaneous drainage, and uterine repair techniques (5, 9–11). However, the optimal techniques remain uncertain, because factors associated with wound complications after CS involve many other aspects, such as nutrition status, age, obesity, diabetes mellitus, anemia, and so on (12).

Wound healing is a systematic process, traditionally explained in terms of three classic phases: inflammation, proliferation, and maturation (13). Adequate amounts of nutrients are needed for synthesis of nucleic acids, proteins, and other factors involved in functional tissue maturation and differentiation (14). Collagen is a natural substrate for cellular attachment, growth, and differentiation and is involved in all three phases of the wound healing cascade. Certain sequences of the collagen fibrils are chemotactic and promote cellular proliferation and differentiation (15).

Collagen-based biomaterials, such as collagen gel, collagen sponge, and collagen dressing, have been reported to have beneficial biological functions on wound healing (15, 16). However, the effect of collagen intake on wound healing is rarely concerned, especially in the case of post-caesarean. Marine collagen peptides (MCPs), enzymatically hydrolyzed from the skin of chum salmon (*Oncorhynchus keta*), are oligopeptide compounds with a molecular mass ranging from 100 to 860 Da (17). Collagen peptide intake has demonstrated beneficial effects on skin health and wound healing (18–21), and our previous studies have shown that MCPs or skin gelatin were able to promote wound healing in normal or diabetic rats (22, 23).

This study investigates the wound healing potential of MCPs, peptide compounds of low molecular weight derived from chum salmon skin via enzymatic hydrolysis, in post-caesarean rats by biomechanical, biochemical, and histological analyses.

## Methods

### Preparation and identification of MCPs

MCPs were derived from the skin of wild-caught chum salmon (average body weight, 1.47 kg), which were donated by CF Haishi Biotechnology Ltd. Co. (Beijing, China). The MCPs were prepared and identified according to a method described previously (17).

In brief, the skin of chum salmon was first homogenized and emulsified in distilled water. Second, at 40°C and pH 8, complex protease was added for 3 h before inactivation and sterile filtration. Lastly, the mixture was made into a powder by spray-drying to produce MCP powder. HPLC (Waters Corp., Milford, MA, USA), LDI-1700 matrix-assisted laser desorption/ionization time-of-flight mass spectrometry, and H835-50 automatic amino acid analyser (Hitachi, Tokyo, Japan) were used to identify the molecular weight distribution and amino acid composition. The results indicated that the molecular weight distribution of MCPs was 100–860 Da. The results of amino acid composition analysis showed that MCPs were rich in Gly>Glu>Pro>Hyp>Asp>Ala>Arg. Further, MCPs were revealed to contain very little or no carbohydrate by negative staining of the polyacrylamide gel with periodic acid-Schiff reagent.

### Animals and treatment

The institutional and national guidelines for the care and use of animals were followed, and all the experimental procedures involving animals were approved by the Animal Ethics Review Committee of Peking University Health Science Center. Ninety-six pregnant Sprague-Dawley rats were obtained from the Department of Animal Service of Peking University Health Science Center at 14 days of gestation. Before the study, all animals were acclimated to the new environment for 5 days, being housed individually in rooms maintained under controlled environmental conditions (12-h light/dark cycle, temperature approximately 25°C) in an approved animal facility, with free access to tap water and AIN-93G diet, which is recommended to support growth, pregnancy, and lactation by the American Institute of Nutrition (24). Rats were randomly assigned to four groups, that is, the vehicle group and 0.125, 0.375, 1.125 g kg^−1^ body weight MCP groups, with 24 rats in each group. The MCPs and vehicle were intragastrically administered every morning after the surgery day.

### CS model

On Day 19 of the pregnancy, the rats were anaesthetized using intraperitoneal sodium pentobarbital (40 mg/kg). The CS was based on the Bowers procedure (25). A 3.0 cm midline laparotomy incision was made in the lower abdomen, and then a 2.0 cm longitudinal incision was made along the antimesenteric border in the midportion of each uterine horn. The rat pups and placentas were gently extruded through the hysterotomy. Afterwards, the uterine incisions were closed with a continuous non-locking 5-0 polyglycolic acid suture, and the deep abdominal fascia as well as peritoneum was closed with 4-0 polyglycolic acid sutures. The skin was closed separately using the same material (26).

### Sampling of skin and uterus

On Days 7, 14, and 21 after the surgery, eight rats from each group were randomly selected, anaesthetized by CO_2_ inhalation and killed by cervical dislocation. The skin wounds and adjoining normal skin were harvested and separated into three equal strips with the wound at midline. These strips were then used for the tensile strength test, hydroxyproline (Hyp), and histological analyses, respectively. The uterus was removed and the left uterine horn was used to measure the bursting pressure, while the right uterine horn was used for Hyp and histological analyses.

### Measurement of skin wound tensile strength and uterine bursting pressure

The skin wound tensile strength and uterine bursting pressure were measured using a pressure transducer and an amplifier-recorder (Pclab-UE Biomedical Signal Acquiring Processing Systems; Beijing MicroStar Technology Development Company Limited, Beijing, China). The 5×10 mm^2^ skin strip was gripped at the wound edge and pulled slowly in the opposite direction until breakage. Tensile strength was calculated by dividing the breaking strength by the cross-sectional area (27). The uterine horns were suture-ligated 1.0 cm distal to the uterine scar to prevent the leakage of perfusion fluid from the fallopian tubes. The horn was opened transversely 1 cm proximal to the scar. A catheter was inserted into the lumen and fixed in place with a suture. The proximal end of the catheter was connected to a 5 ml injector, and the pressure transducer was connected by a three-way valve. The pressure transducer was connected to the amplifier-recorder. Normal saline was infused into each horn slowly until the horn burst and leakage occurred. The highest pressure that was obtained was designated the bursting pressure (24).

### Quantitative analysis of Hyp

The skin and uterine samples collected for Hyp content assay were trimmed into rectangular pieces, about 20 mg each. Hyp concentration was measured by a chemical colorimetric method using a commercial detection kit (A030 Hydroxyproline Detection Kit; Nanjing Jiancheng Bioengineering Institute, Nanjing, China).

### Histological analysis

The skin and uterine specimens in the vehicle-treated group and the 1.125 g kgbw^−1^ MCP-treated group were collected vertically from the longitudinal scar. Then, specimens were fixed in 10% neutral-buffered formalin for at least 24 h, followed by processing for conventional paraffin embedding. Sections measuring 5 µm thick were mounted on glass slides, dewaxed, rehydrated to distilled water, and stained with hematoxylin and eosin or Masson's trichrome. Morphological findings, including fibroblast proliferation, vascularization, and collagen formation, were semi-qualitatively assessed under the light microscope using a four-point scale as follows: ‘none’, 0; ‘few’, 1; ‘moderate’, 2; and ‘many’, 3. Two independent pathologists performed the histological examination and applied the scoring system in a blinded fashion (28, 29).

### Immunohistochemistry

Paraffin-embedded tissues were sectioned (5 µm), and antigen retrieval was performed using 10 mM sodium citrate buffer. Endogenous peroxidase activity was blocked by treating sections with 0.3% hydrogen peroxide in methanol for 15 min. Tissues were treated with polyclonal rabbit anti-transforming growth factor beta 1 (TGF-β1), anti-basic fibroblast growth factor (bFGF), and anti-platelet-endothelial cell adhesion molecule-1 (CD31) antibody (Santa Cruz Biotechnology, Inc., Santa Cruz, CA, USA; dilution 1:300) overnight at 4°C. Specific labelling was detected with a peroxidase-conjugated goat anti-rabbit IgG and avidin-biotin peroxidase complex. Slides were then mounted with coverslips and analyzed by two blinded pathologists. The expression of TGF-β1 and bFGF was assessed by immunoreactive cell density plus expression intensity. Immunoreactive cell density was graded semi-quantitatively as 1) less than 10% per field, 2) 10–30% per field, 3) 30–70% per field, or 4) more than 70% per field, whereas expression intensity was graded as 1) mild, 2) moderate, or 3) severe. At the same time, the number of microvessels was counted to assess CD31 expression. All assessments were performed in a blind manner by two investigators, and five regions of interest in each specimen were used for each determination.

### Statistical analysis

Data were expressed as means and standard deviations, and statistical significance between the experimental and control values was analyzed using one-way ANOVA test, except for the data obtained from the semi-quantitative analysis, which were analyzed using the Mann–Whitney U test. The software SPSS 18.0 (SPSS, Inc., Chicago, IL, USA) was used, and a *p*-value ≤0.05 was considered statistically significant.

## Results

### General information

The body weight, uterine weight, waistline, and food consumption among the groups did not show any significant difference on Days 7, 14, and 21. Based on the food consumption and peptides administered by gavage, the energy and protein consumption were calculated and compared. There were no remarkable differences among the groups. Similarly, no significant differences in the adhesion levels of the uterine incision in the fatty or abdominal tissue, were observed between the MCP-treated groups and the vehicle group (data not shown). The average time for wound healing was 6–7 days. The healing time for skin wounds in the MCP groups was 1–2 days less than the control.

### Tensile strength and uterine bursting pressure

**Fig. 1:**
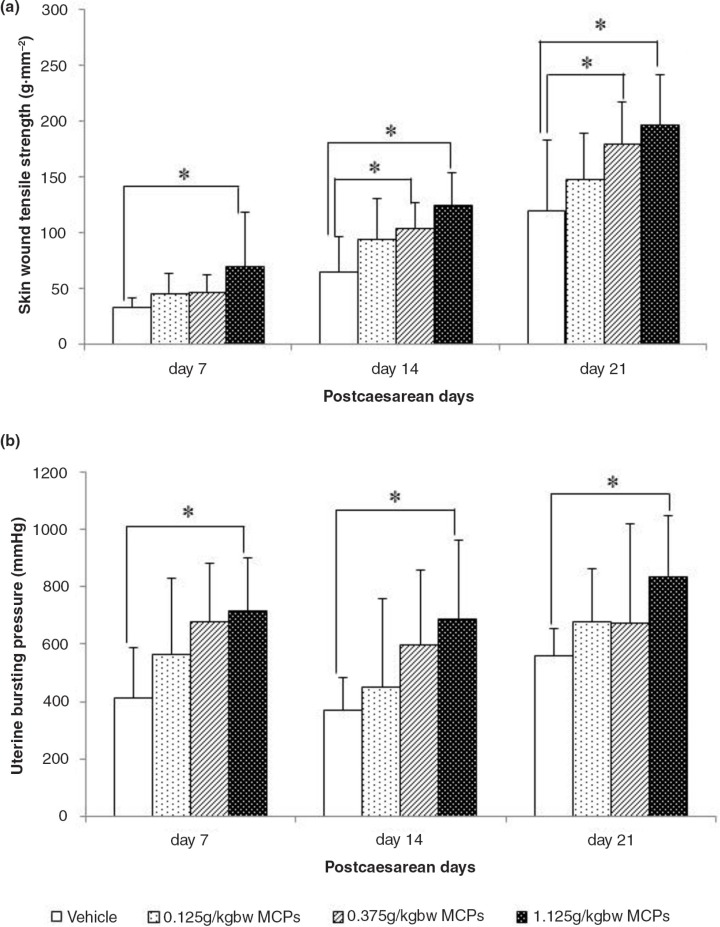
(a) Skin wound tensile strength measured on day 7, 14 and 21 from vehicle and MCP-treated rats. (b) Uterine bursting pressure measured on day 7, 14 and 21 from vehicle and MCP-treated rats. MCPs, marine collagen peptides. Values are presented as mean±SD, *n*=8 for each group. *Significant difference at *P*<0.05.

The skin wound tensile strength and uterine bursting pressure continued to increase from 7 to 21 days after CS surgery. Our results showed that the skin wound tensile strength of rats in the 1.125 g kgbw^−1^ MCP-treated group was significantly higher than that in the vehicle-treated group on Days 7, 14, and 21 post-caesarean, respectively (*p*<0.05), whereas the same increase was also found in the 0.375 g kgbw^−1^ MCP-treated group on Days 14 and 21 (Fig. 1a). Moreover, we also indicated a dose-dependent effect of MCPs on tensile strength; the linear trend model test showed that the *p*-values were 0.017, 0.005, and 0.001 on Days 7, 14, and 21, respectively. The uterine bursting pressure of the rats in the 1.125 g kgbw^−1^ MCP group also increased remarkably at all three time points (Fig. 1b). In addition, a dose-response relationship was observed in groups, with *p*-values of 0.005, 0.009, and 0.029 on Days 7, 14, and 21, respectively.

### Histological analysis and quantitative analysis of Hyp

#### Histological analysis

**Fig. 2:**
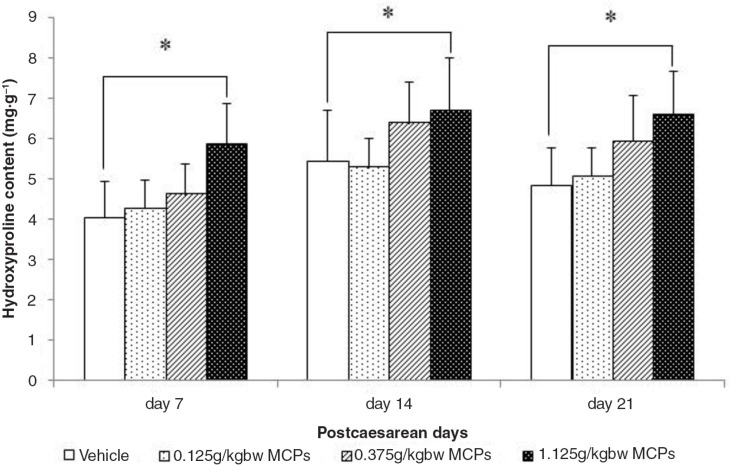
Hydroxyproline levels in the skin wound tissue from vehicle and MCP-treated rats at days 7, 14 and 21. Values are presented as mean±SD, *n*=8 for each group. *Significant difference at *P*<0.05.

Hyp levels both in skin wound tissue and uterine wound tissue were measured at 7, 14, and 21 days after CS. The results showed that Hyp levels in skin wound tissue in the 1.125 g kgbw^−1^ MCP-treated group were higher than those in the vehicle-treated group (Fig. 2) and significant differences were observed at all three time points (*p*<0.05). Also, the linear trend model test showed that there was a dose-dependent effect of MCPs on Hyp levels, with the *p*-values being 0.001, 0.039, and 0.004 on Days 7, 14, and 21, respectively. However, no significant differences on Hyp levels were observed in uterine scar tissue (data not shown for sample).

#### Quantitative analysis of Hyp

**Fig. 3:**
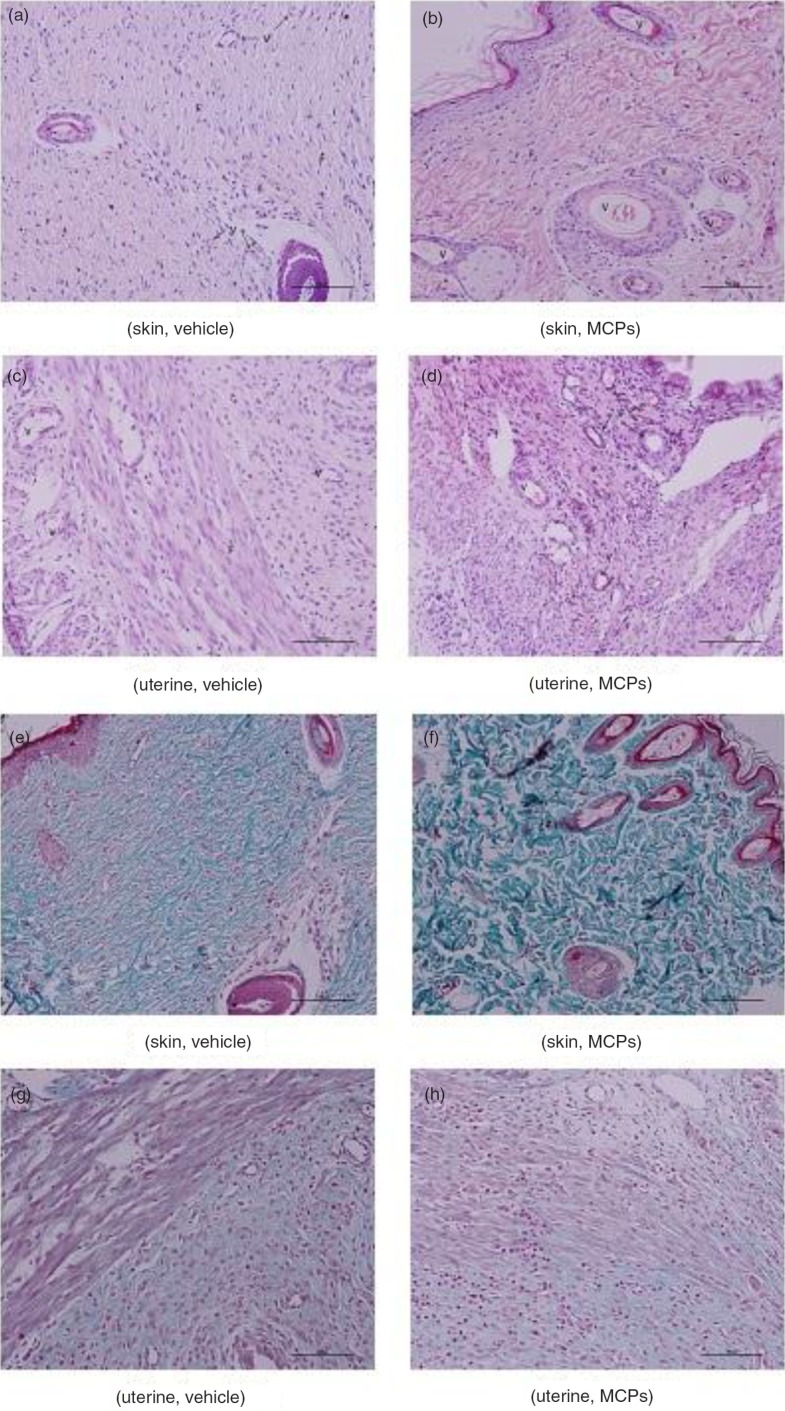
(a–d) Representative micrograph of hematoxylin and eosin-stained section of both skin and uterine wounds treated with vehicle and 1.125 g kg^−1^ MCPs at day 7. (e–h) Masson-stained section of both skin and uterine wounds treated with vehicle and 1.125 g kg^−1^ MCPs at day 21. (Magnification, 5×40).

The degree of fibroblast proliferation, vascularization, and collagen formation, both in the skin wound area and in the uterine wound area, is shown in Table 1. Compared with the vehicle-treated group, rats in the 1.125 g kgbw^−1^ MCP group tended to demonstrate increased vascularization in both skin and uterine wound tissue (Fig. 3a through d). Vascularization both in skin and uterine tissue were significantly increased in the 1.125 g kgbw^−1^ MCP-treated group compared to the vehicle-treated group at 7 days (*p*<0.05) (Table 1). Furthermore, as shown in Table 1 and Fig. 3e through h, significant collagen deposition was observed in skin wound tissue in the 1.125 g kgbw^−1^ MCP-treated group at 14 and 21 days post-caesarean (*p*<0.05).

**Table 1:** Histological score of skin and uterine wound tissue (*n*=4)

	Skin wound tissue			Uterine wound tissue		
		
Day/sample	Fibroblasts	Vascularization	Collagen	Fibroblasts	Vascularization	Collagen
Day 7						
Vehicle	0.88±0.84	0.75±0.46	1.13±0.35	0.75±0.71	0.50±0.54	0.63±0.54
1.125 g·kgbw^−1^ MCPs	1.25±0.71	2.00±0.76*	1.25±0.71	1.25±1.04	1.50±0.76*	0.75±0.46
Day 14						
Vehicle	1.88±0.84	1.63±0.52	1.50±0.76	1.50±0.54	0.88±0.64	0.88±0.84
1.125 g·kgbw^−1^ MCPs	1.88±0.64	2.00±0.76	2.38±0.74*	1.63±0.52	1.50±0.54	1.13±1.13
Day 21						
Vehicle	2.25±0.71	1.00±0.76	1.63±0.52	1.63±0.52	0.38±0.52	1.13±0.84
1.125 g·kgbw^−1^ MCPs	2.50±0.54	1.00±0.76	2.50±0.54*	1.75±0.71	0.75±0.71	1.38±1.06

Histological scorings were performed in two groups: vehicle-treated group and 1.125 g·kg^−1^ MCP-treated group. Data were expressed as mean±SD.

* *P*<0.05, compared with vehicle-treated group.

### Immunohistochemistry

The TGF-β1, bFGF, and CD31 immunoreactivity of skin and uterine wound sections were examined in the 1.125 g kg^−1^ MCP- and vehicle-treated groups at 7, 14, and 21 days post-caesarean. The numbers of cells expressing TGF-β1, bFGF, and CD31 were counted. As shown in Table 2, in skin wound tissue, TGF-β1 expression was significantly higher in the MCP-treated groups than in the vehicle-treated group at Days 14 and 21 post-caesarean (*p*<0.05); bFGF expression was significantly higher in the MCP-treated groups than in the vehicle-treated group at Days 7 and 14 post-caesarean (*p<*0.05); whereas CD31 expression was significantly higher in the MCP-treated groups than in the vehicle-treated group at Day 7 post-caesarean (*p<*0.05). However, we did not observe any significant difference in TGF-β1, bFGF, and CD31 expression between the vehicle- and MCP-treated groups in uterine wound tissue.

**Table 2:** Immunohistochemistry assessment of TGF-β1, bFGF, and CD31 expression in skin and uterine wound tissue (*n*=4)

	Skin wound tissue			Uterine wound tissue		
		
Day/sample	TGF-β1	bFGF	CD31	TGF-β1	bFGF	CD31
Day 7						
Vehicle	3.25±1.07	2.65±1.09	15.15±2.70	2.20±1.10	2.60±1.05	13.60±2.72
1.125 g·kgbw^−1^ MCPs	3.70±0.98	4.75±1.33*	20.40±3.76*	2.55±1.05	3.00±1.02	14.65±3.15
Day 14						
Vehicle	2.95±1.00	3.15±1.14	10.60±2.08	2.05±1.05	2.60±0.88	10.45±2.33
1.125 g·kgbw^−1^ MCPs	5.30±1.75*	3.90±0.97*	11.70±3.58	2.40±1.19	2.90±1.16	11.60±3.42
Day 21						
Vehicle	2.50±1.00	3.60±1.14	9.75±1.86	2.15±0.81	2.75±0.91	9.35±2.01
1.125 g·kgbw^−1^ MCPs	4.15±1.14*	4.05±0.89	10.40±2.58	2.70±1.22	3.00±1.02	10.45±3.53

Immunohistochemistry assessment was performed in two groups: vehicle-treated group and 1.125 g·kg^−1^ MCP-treated group. Data were expressed as mean±SD.

* *P*<0.05, compared with vehicle-treated group.

## Discussion

The goal of the present study is to reveal whether or not oral administration of MCPs accelerates wound healing following cesarean section in rats.

Wound healing is complex and involves multiple molecular processes, including inflammation, angiogenesis, granulation tissue formation, re-epithelialization, and wound contraction (23, 30). Therefore, an effective treatment that improves wound healing is unlikely to involve only one or two of these components. In order to determine the effects of MCPs on the different phases of wound healing, we collected tissue samples at Days 7, 14, and 21 post-caesarean.

The tensile strength is the most common parameter in the evaluation of wound healing, both in skin and uterine tissue (24, 27). Because the tensile strength of scar tissue is much weaker than that of normal tissue, a higher tensile strength reflects a faster healing process. The skin wound tensile strength and uterine bursting pressure kept increasing from 7 to 21 days after CS surgery. Meanwhile, there is a correlation between CS and the risk of uterine rupture during pregnancy: As evidenced by previous studies, women who had a CS in a previous pregnancy have an almost 10-fold greater risk of uterine rupture than those with an intact uterus (31). Therefore, we assume that the healing of uterine wounds can influence the risk of rupture and that a uterus that has healed soundly can decrease the risk of rupture. In this study, it was demonstrated by a remarkable increase in the skin tensile strength and uterine bursting pressure.

Complete wound healing is the result of a complex set of interactions between cellular and acellular components. Microscopically, this process possesses newly formed capillaries and fibroblasts. This study demonstrated that histopathological evaluation of the wound site provided evidence of a more desirable histological organization of the tissue in response to treatment with MCPs.

Angiogenesis, the formation of new blood vessels, is essential to the wound healing process. Immediately after wounding, it allows delivery of oxygen, nutrients, and inflammatory cells to the site of injury. It also assists in granulation tissue formation and ultimately wound closure. Compared with the vehicle-treated group, rats in the 1.125 g kgbw^−1^ MCP group tended to demonstrate increased vascularization in both skin and uterine wound tissue.

Furthermore, collagen is required for wound healing. Several studies reported an increase in wound tensile strength dependent on factors in addition to collagen deposition, namely matrix deposition and cell migration (32). Stimulating their synthesis when wound repair is defective would be beneficial for promoting wound healing. Significant collagen deposition, measured as Masson staining, was observed in skin wound tissue in the 1.125 g kgbw^−1^ MCP-treated group at 14 and 21 days post-caesarean. Initially, wounds have observably minimal breaking strength because the clot alone holds the edges together (33, 34). Thereafter, tensile strength increases rapidly as collagen deposition increases and cross linkages are formed between the collagen fibers. Moreover, treatment of rats with MCPs resulted in an enhancement of wound healing, as evidenced by the increased skin tensile strength and uterine bursting pressure. Approximately 99.8% of the body's stores of Hyp are found in collagen, which renders assays of this amino acid useful as a marker for the total amount of collagen present (35). Hyp is a derivative of proline; both proline and Hyp are vital for collagen biosynthesis, structure, and strength. Dietary proline is necessary for promoting tissue repair in both animals and humans with wounds and burns (36). Similar results have been obtained in this study. In this study, Hyp levels both in skin wound tissue and uterine wound tissue were measured at 7, 14, and 21 days after CS. The results showed that Hyp levels in skin wound tissue in the 1.125 g kgbw^−1^ MCP-treated group were higher than those in the vehicle-treated group, and significant differences were observed at all three time points. Also, the linear trend model test showed that there was a dose-dependent effect of MCPs on Hyp levels. However, previous studies reached different conclusions regarding the meaning of collagen content in uterine tissue (24, 37, 38).

Fortunately, wound healing, like all inflammation-based processes in the body, is based on various redundant signals and cross communication between different signaling networks (39). TGF-β released from platelets and leukocytes is a multifunctional cytokine that plays an important role in cell migration, proliferation, differentiation, apoptosis, and extracellular matrix (ECM) protein production (40–43). During inflammation, TGF-β is crucial for guiding the recruitment of fibroblasts and endothelial cells (ECs) later in the healing process. TGF-β1, a member of the TGF superfamily, is a potent profibrotic factor that strongly induces collagen synthesis (44). There are indications that MCPs might improve the synthesis of collagen in proliferation and maturation, by enhancing the expression of TGF-β1. At the same time, bFGF is a potent mitogen and a modulator for fibroblasts and vascular ECs (45) and plays an important role in tissue regeneration and repair (46). Within hours of injury, re-epithelialization is initiated, and bFGF is released to stimulate epithelial cell migration and proliferation. This process begins with the dissolution of cell–cell and cell–substratum contacts, followed by polarization and migration of keratinocytes over the provisional ECM. In the present study, it is indicated that MCPs might improve the synthesis of ECM components in re-epithelialization and maturation, by enhancing the expression of bFGF. In addition, CD31 (PECAM-1) is a 130-kDa transmembrane glycoprotein that is a member of the Ig superfamily of cell adhesion molecules and is expressed on EC as several circulating blood elements including platelets, polymorphonuclear leukocytes, monocytes, and lymphocytes (47). Due to its expression on vascular and hematopoietic cells and its signaling and adhesive capabilities, CD31 is primed to serve a vital role in inflammation. Indeed, it is indicated that CD31 as both a positive regulator in cute pro-inflammatory and negative regulator of chronic inflammatory responses (48). As Day 7 was the end of the inflammation period, it meant that MCPs might alleviate inflammation by up-regulating the expression of CD31.

## Conclusions

In conclusion, oral MCPs administration improves wound healing following CS in rats by enhancing wound contraction, collagen deposition and angiogenesis. Taken together, we believe that oral MCPs are therapeutically beneficial method to treat wounds in clinical practice.

## References

[1] Tang SL, Li XY, Wu ZC (2006). Rising cesarean delivery rate in primiparous women in urban China: evidence from three nationwide household health surveys. Am J Obstet Gynecol.

[2] Klemetti R, Che X, Gao Y, Raven J, Wu Z, Tang S (2010). Cesarean section delivery among primiparous women in rural China: an emerging epidemic. Am J Obstet Gynecol.

[3] Villar J, Valladares E, Wojdyla D, Zavaleta N, Carroli G, Velazco A (2006). Caesarean delivery rates and pregnancy outcomes: the 2005 WHO global survey on maternal and perinatal health in Latin America. Lancet.

[4] Lumbiganon P, Laopaiboon M, Gülmezoglu AM, Souza JP, Taneepanichskul S, Ruyan P (2010). Method of delivery and pregnancy outcomes in Asia: the WHO global survey on maternal and perinatal health 2007–08. Lancet.

[5] Clay FS, Walsh CA, Walsh SR (2011). Staples vs subcuticular sutures for skin closure at cesarean delivery: a meta-analysis of randomized controlled trials. Am J Obstet Gynecol.

[6] Owen J, Andrews WW (1994). Wound complications after cesarean sections. Clin Obstet Gynecol.

[7] Naumann RW, Hauth JC, Owen J, Hodgkins PM, Lincoln T (1995). Subcutaneous tissue approximation in relation to wound disruption after cesarean delivery in obese women. Obstet Gynecol.

[8] Yazicioglu F, Gökdogan A, Kelekci S, Aygün M, Savan K (2006). Incomplete healing of the uterine incision after caesarean section: is it preventable?. Eur J Obstet Gynecol Reprod Biol.

[9] Hofmeyr JG, Novikova N, Mathai M, Shah A (2009). Techniques for cesarean section. Am J Obstet Gynecol.

[10] Hellums EK, Lin MG, Ramsey PS (2007). Prophylactic subcutaneous drainage for prevention of wound complications after cesarean delivery: a meta-analysis. Am J Obstet Gynecol.

[11] Walsh CA, Walsh SR (2009). Extra-abdominal vs intra-abdominal uterine repair at cesarean delivery: a meta-analysis. Am J Obstet Gynecol.

[12] Gall SA, Phelan JP, Clark SL (1988). Diagnosis and management of postcesarean wound complications. Cesarean delivery.

[13] Baum CL, Arpey CJ (2005). Normal cutaneous wound healing: clinical correlation with cellular and molecular events. Dermatol Surg.

[14] Brown KL, Phillips TJ (2010). Nutrition and wound healing. Clin Dermatol.

[15] Ruszczak Z (2003). Effect of collagen matrices on dermal wound healing. Adv Drug Deliv Rev.

[16] Fleck CA, Simman R (2010). Modern collagen wound dressings: function and purpose. J Am Col Certif Wound Spec.

[17] Pei X, Yang R, Zhang Z, Gao L, Wang J, Xu Y (2010). Marine collagen peptide isolated from chum salmon (*Oncorhynchus keta*) skin facilitates learning and memory in aged C57BL/6J mice. Food Chem.

[18] Zague V (2008). A new view concerning the effects of collagen hydrolysate intake on skin properties. Arch Dermatol Res.

[19] Zague V, Freitas V, Rosa MC, de Castro GÁ, Jaeger RG, Machado-Santelli GM (2011). Collagen hydrolysate intake increases skin collagen expression and suppresses matrix metalloproteinase 2 activity. J Med Food.

[20] Tanaka M, Koyama Y, Nomura Y (2009). Effects of collagen peptide ingestion on UV-B-induced skin damage. Biosci Biotechnol Biochem.

[21] Demidova-Rice TN, Geevarghese A, Herman IM (2011). Bioactive peptides derived from vascular endothelial cell extracellular matrices promote microvascular morphogenesis and wound healing *in vitro*. Wound Repair Regen.

[22] Zhang Z, Zhao M, Wang J, Ding Y, Dai X, Li Y (2011). Oral administration of skin gelatin isolated from chum salmon (*Oncorhynchus keta*) enhances wound healing in diabetic rats. Mar Drugs.

[23] Zhang Z, Wang J, Ding Y, Dai X, Li Y (2011). Oral administration of marine collagen peptides from Chum Salmon skin enhances cutaneous wound healing and angiogenesis in rats. J Sci Food Agric.

[24] Bowers D, McKenzie D, Dutta D, Wheeless CR, Cohen WR (2001). Growth hormone treatment after cesarean delivery in rats increases the strength of the uterine scar. Am J Obstet Gynecol.

[25] Reeves PG, Nielsen FH, Fahey GJ (1993). AIN-93 purified diets for laboratory rodents: final report of the American Institute of Nutrition ad hoc writing committee on the reformulation of the AIN-76A rodent diet. J Nutr.

[26] Gul A, Kotan C, Ugras S, Alan M, Gül T (2001). Transverse uterine incision non-closure versus closure: an experimental study in dogs. Eur J Obstet Gynecol Reprod Biol.

[27] Eroglu E, Eroglu F, Agalar F, Altuntas I, Sutcu R, Ozbasar D (2001). The effect of lidocaine/prilocaine cream on an experimental wound healing model. Eur J Emerg Med.

[28] Qiu Z, Kwon AH, Kamiyama Y (2007). Effects of plasma fibronectin on the healing of full-thickness skin wounds in streptozotocin-induced diabetic rats. J Surg Res.

[29] Wilgus TA, Vodovotz Y, Vittadini E, Clubbs EA, Oberyszyn TM (2003). Reduction of scar formation in full-thickness wounds with topical celecoxib treatment. Wound Repair Regen.

[30] Jacobs S, Simhaee DA, Marsano A, Fomovsky GM, Niedt G, Wu JK (2008). Efficacy and mechanisms of vacuum-assisted closure (VAC) therapy in promoting wound healing: a rodent model. J Plast Reconstr Aesthet Surg.

[31] Locatelli A, Ghidini A, Ciriello E, Incerti M, Bonardi C, Regalia AL (2006). Induction of labor: comparison of a cohort with uterine scar from previouscesarean section vs. a cohort with intact uterus. J Matern Fetal Neonatal Med.

[32] Achuth HN, Moochhala SM, Mahendran R, Tan WT (2005). Nitrosoglutathione triggers collagen deposition in cutaneous wound repair. Wound Repair Regen.

[33] Naik HR, Naik HS, Naik TR, Naika HR, Gouthamchandra K, Mahmood R, Ahamed BM (2009). Synthesis of novel benzo[h]quinolines: wound healing, antibacterial, DNA binding andin vitro antioxidant activity. Eur J Med Chem.

[34] Lodhi S, Pawar RS, Jain AP, Singhai AK (2006). Wound healing potential of *Tephrosia purpurea* (Linn.) Pers. in rats. J Ethnopharmacol.

[35] Perez GR, Vargas SR, Ortiz HY (2005). Wound healing properties of *Hylocereus undatus* on diabetic rats. Phytother Res.

[36] Barbul A (2008). Proline precursors to sustain mammalian collagen synthesis. J Nutr.

[37] Pettersson KW, Grunewald C, Thomassen P (2007). Uterine rupture and perinatal outcome. Acta Obstet Gyn Scan.

[38] Buhimschi CS, Buhimschi IA, Yu CL, Wang H, Sharer DJ, Diamond MP (2006). The effect of dystocia and previous cesarean uterine scar on the tensile properties of the lower uterine segment. Am J Obstet Gynecol.

[39] Vodovotz Y, Csete M, Bartels J, Chang S, An G (2008). Translational systems biology of inflammation. Comput Biol.

[40] Annes JP, Munger JS, Rifkin DB (2003). Making sense of latent TGFβ activation. J Cell Sci.

[41] Baudino TA, Carver W, Giles W, Borg TK (2006). Cardiac fibroblasts; friends or foe?. Am J Physiol Heart Circ Physiol.

[42] Hinck AP (2012). Structural studies of the TGF-βs and their receptors – insights into evolution of the TGF-β superfamily. FEBS Lett.

[43] Sales VL, Engelmayr GC, Mettler BA, Johnson JA, Sacks MS, Mayer JE (2006). Transforming growth factor-β1 modulates extracellular matrix production, proliferation, and apoptosis of endothelial progenitor cells in tissue-engineering scaffolds. Circulation.

[44] Wynn TA (2008). Cellular and molecular mechanisms of fibrosis. J Pathol.

[45] Squires CH, Childs J, Eisenberg SP, Polverini PJ, Sommer A (1988). Production and characterization of human basic fibroblast growth factor from *Escherichia coli*. J Biol Chem.

[46] Tabata Y, Ikada Y (1999). Vascularization effect of basic fibroblast growth factor released from gelatin hydrogels with different biodegradabilities. Biomaterials.

[47] Smpio BE, Yun S, Cordova AC, Haga M, Zhang J, Koh Y (2005). MAP kinase (ERKl/2, p38) and AKT can be phosphorylated by shear stress independently of PEC AM-1 (CD31) in vascular endothelial cells. J Biol Chem.

[48] Jamie RP, Debra KN, Peter JN (2010). PECAM-1: conflicts of interest in inflammation. Life Sci.

